# The Relations and Resources of Our Specialty

**Published:** 1891-09

**Authors:** Horatio C. Meriam

**Affiliations:** Salem, Mass.


### THE RELATIONS AND RESOURCES OF OUR SPECIALTY.[1]

BY HORATIO C. MERIAM, D.M.D., SALEM, MASS.

To the Greeks, *Physic* meant Nature, and *Physician* Naturalist. These definitions are not without value to us now, when in the evolution or growth of our specialty there seems to be developing a class of practitioners who, basing their practice on a study of nature, her laws and forms, work for their preservation and restoration, thus enlarging the scope and range of our specialty, so that where we formerly heard of a fine operator, we now hear of a good adviser, and in place of resting reputation on a fine filling or a "good job," we say that such a space was well treated, the arch well preserved, or that the mouth showed good care. Thus we have practitioners who draw their theories from nature, leaving behind that other class of practitioners who draw their patients to suit their theories.

Nor are the former less free because free within nature's limits. For the acceptance of her as a guide and teacher implies that, in order to follow this guidance and teaching, they are to be free from many of the restraints that hamper and limit commercial pursuits. The professional man thus following and working can say, in the language of the Epistle to the Phillippians, "Not as though I had already attained, either were already perfect: but I follow after, if that I may apprehend that for which also I am apprehended. . . ."

There goes with this, of course, freedom to follow, that Truth may be found and shown to other men, and as it is freedom to serve in a cause that is greater than self, of such a professional spirit it may be said,—

> "How haply is he born or taught
> Who serveth not another's will;
> Whose armor is his honest thought,
> And simple truth his utmost skill."

This would seem to meet an objection that I have met among those who have not studied ethics,—*i.e.*, "*our acts, their motives and tendencies*," as they have been studied in this Society. It was said to me, "You do not allow your patients to dictate to you, and why should you expect to dictate to those who supply you?" "On the contrary," I replied, "every patient dictates to us. Every patient comes to us with certain conditions of the mouth, the teeth, or the

---

[1] Read before the New York Odontological Society, June 16, 1891.

functions of which they form a part, that dictate to us, and our reputations are made by the clearness with which we read those conditions and the ability with which we bring the resources of our specialty to meet them." They thus must needs have all the resources of nature and of art as an armamentarium placed at their disposal, who are to defend the temple whose builder and maker is God. Thus the physician or "naturalist" called to treat the eye, the ear, the teeth, the crooked limb, or the weary brain, accepts the dictation of their condition, and in accepting secures the right to dictate how the disease or deformity shall be met.

It may be some Bigelow consulted regarding the hip or stone in the bladder, some Bowditch regarding the lungs, but whosoever the man or whatever the disease, all the claims of humanity assert his right and his liberty to prescribe to meet disease as he finds it. Shall this workman lose his right hand? Still the surgeon's prescription, though it is to be done by the hand. Shall this patient leave home and family and live in another climate? still a prescription. Shall this arch be enlarged or reduced? This foot twisted or cut? Whatever the case, whatever the remedy or treatment indicated,

> ". . . the odors of myrtle or pine,
> Breeze of the prairie or breath of the sea,"

it still remains a prescription.

So we should assert that with us rests the right of prescribing for our patients' needs, and the dictation, or direction, if you like it better, as to how those needs shall be meet.

We know that every thought or action represents destruction. The sermon of the ecclesiastic, the song of the poet, the sigh of the lover, the cheer of the patriot, all represent the destruction of a certain number of cells, whose destruction has set force free. Even holding the faith that saves or the doubts that distress calls for a physical system that must be continually undergoing destruction and repair, and as growth or action is founded on the destruction of previous growth, new cells being constantly produced to replace those used, we have "the sum of all action,—life."

A specialty having care of a great and important function, one which is so essentially connected in supplying the needs of all other functions that make a complete life, must have a growth as great as the needs to which it ministers, and resources as great to meet those needs.

We have heard the claim that "our specialty owes all it is to its instrument-makers, and that they have only to cease making for a time to insure our destruction," and the other claim that "they own so many patents that they can take three millions a year from our specialty where they take one now." Still, as there was much in the world before we came, and, strange to say, much will be left in it after we leave, I am inclined to think that they take themselves a little too seriously and that the great and terrible day of an instrument-maker's wrath will not come to us this year. We have but to open our eyes to the great resources around us, and connect our specialty more intimately with them.

"You remember how, when the city was besieged, each artisan who was called upon in council to suggest the best means of defence recommended the articles he dealt in: the carpenter, wood; the blacksmith, iron; the mason, brick; until it came to be a puzzle to know which to adopt. Then the shoemaker said, 'Hang your walls with new boots.'"

Now, all these things are needed in the defence of the city, though perhaps not quite in the way that the dealers proposed; the wood, the iron, the bricks, the boots, would all be of service, but there was needed some one to prescribe for each its fitting place.

We may perhaps feel that the English makers in consenting to advance the price of teeth in England lost all claim to special sympathy; nevertheless, we resent as an interference any attempt to exclude from American practice anything required by us in meeting the needs of our patients.

We should give our workmen all the credit due, payment and praise also, but not the power to dictate regarding what shall be used in our practice. Praise when they do that which is right and make that which is good, but make clear also that they are to make that which we think is good for the needs of our patients, and for the development of our specialty.

They might build works as great as the Pyramids of Egypt, and build them well, but if they were not what we wished to prescribe, or to use in making our prescriptions, they would be of interest only as monuments of power or bigness.

"If they be not big for me,
What care I how big they be?"

There will, of course, be a great difference in the development and position of those practitioners for whom makers make that which they wish to use, and the other class of practitioners who are obliged to use those things that makers wish to make.

Perhaps *development* should have been included in the title of this address, for the effect of controlling our relations and resources will have much to do with the development of our specialty.

We need the increased skill of the hand, secured by creating what the brain has conceived; and as the hand and brain are developed by reciprocal action, we get increased brain-power with increasing skill of the hand.

The business of the busy practitioner is not to invent new resources or instruments for their own sake, but to adapt resources to patients.

Some of the finest development in our specialty is seen in connection with the ability to use the old, but there are some whose development we should have greatly missed had they not worked out their conceptions, and given them to us in forms of beauty and marvellous adaptability.

Just as the same blood builds and nourishes the foot that walks and the brain that thinks, so we do not feel that anything can be held as not included in medicine because mechanical or of use in other ways.

"Pressure produces absorption" is a law alike in orthopedics and in orthodontia. Should a physician refuse to use nitre because it is also used in the arts, in gunpowder, and in agriculture as a fertilizer? We cannot feel that anything that can affect our specialty in any way is not to be studied in connection with it, or can be rightly studied as isolated from "nature," "medical science," or "the healing art," but by keeping clear to ourselves and our students that our work is a part of a system for the making the best of our race. We connect it with interests as great and far-reaching as any of the problems of life. "Is there anything better in a State," says Plato, "than that women and men be rendered the very best?" Violators of nature can never be held in high esteem. It is quoted from Shakespeare, that, "One said a tooth-drawer was a kind of unconscionable trade, because his trade was nothing else but to take away those things whereby every man gets his living."

I beg to quote somewhat at length from Dr. Holmes's "Medical Essays," this broad definition and description of medicine.

"Medicine, sometimes impertinently, often ignorantly, often carelessly called 'allopathy,' appropriates everything, from every source, that can be of the slightest use to anybody who is ailing in any way, or like to be ailing from any cause. It learned from a monk how to use antimony, from a Jesuit how to cure agues, from a friar how to cut for stone, from a soldier how to treat gout, from a sailor how to keep off scurvy, from a postmaster how to sound the Eustachian tube, from a dairy-maid how to prevent small-pox, and from an old market-woman how to catch the itch-insect. It borrowed acupuncture and the moxa from the Japanese heathen, and was taught the use of lobelia by the American savage. It stands ready to-day to accept anything from any theorist, from any empiric, who can make out a good case for his discovery or his remedy. 'Science' is one of its benefactors, but only one out of many. Ask the wisest practising physician you know what branches of science help him habitually, and what amount of knowledge relating to each branch he requires for his professional duties? He will tell you that scientific training has a value independent of all the special knowledge acquired. He will tell you that many facts are explained by studying them in the wider range of related facts to which they belong. He will gratefully recognize that the anatomist has furnished him with indispensable data, that the physiologist has sometimes put him on the track of new modes of treatment, that the chemist has isolated the active principles of his medicines, has taught him how to combine them, has from time to time offered him new remedial agencies, and so of others of his allies."

Our specialty can assert that we have a claim on all people that have the best at heart; that just as it is the duty of the good citizen to be interested in government, churches, schools, science, the administration of justice, and maintaining the public health, it is likewise their duty to see that our specialty is unhampered, that we are not isolated, but make a part of a great system that helps men and women to be and to do their best.

Life has become so complex and interwoven that the competent man has much put on him to do, and, like the locomotive, must be kept in the best order possible to do it. Still comfort breeds forgetfulness; but the members of a community do not discharge all their obligation to our specialty in paying the bill of their own dental attendant.

Our schools should not be isolated, but become known as correlated with educational institutions. A wise course has in the past few years been adopted by the Harvard Dental School of making more of her position as part of the University, and thus securing the attention to the work she is doing for the good of the community, and, as the world always helps those that she can see are helping others, the fund for the school's endowment has grown more since this policy was adopted than in all the previous years.

The advantage to be gained by having our schools advertised in educational journals, especially in that we thus reach teachers, should not be overlooked; for many bright men often teach before selecting a profession, and we shall also extend the relations of our specialty.

Let us as far as possible cease to be exclusive, learn to be liberal and connected as we can be with the world around us. It was not pleasant some years ago, on going into a shop that supplies materials and apparatus to the Institute of Technology, the medical schools, and other educational institutions, to find that they did not know of the dental schools.

It is a good rule to trade with those who can consult you in return, and, being known to all makers and dealers in articles we can use, we shall secure wider relations for our specialty and increased employment for ourselves.

During my service in the Massachusetts Dental Society, I found that had I been practising in Boston my acquaintance and practice would have been increased by the wider contact gained in helping secure exhibitors, and we should all gain by scattering our purchases where they would do us the most good. Every dental school can secure a directory of all who can make or deal in any of our resources, not only for the economy in purchasing, but for the increased contact with the world and the increased influence for them and their students that they will gain.

The same course should be pursued in seeking advertisements for our journals. Many of the dealers in small tools and various articles we use, sell, I have been informed, many thousands' worth in a year, that come to us through the "depots." And by securing advertisements of such dealers we shall gain increased influence and profit for our journals.

One of the disadvantages of the combination and its control of journals seems to be, that in order to prevent competition, by limiting the number of those who can engage in dealing in our resources, it must strive to prevent a knowledge of their source and relations.

In addition to my dental journals I take the *Druggists' Circular*, 72 William Street, New York City, which, besides giving many valuable articles on medicines and chemistry, usually has a number of pages giving the composition and directions for making many preparations, both professional and proprietary.

Its advertising pages represent a wide constituency, too numerous to mention here, but which if followed will show much of value to us in materia medica. The criticisms of chemists and pharmacists on antiseptics are of special value, but the study of our relations and resources will lead us into many fields.

Thus wheels, brushes, buffing-felt, etc., belong with polishing supplies. I heard of one firm in a neighboring city, which in 1890 cut over one million two hundred thousand yards of cloth into buffing-wheels. The Chicago Watch-Tool Company, of Chicago, the Faneuil Watch-Tool Company, of Boston, the American Watch-Tool Company, of Springfield, Mass., and many others make polishing wheels and lathes. Bottles, lamps, tooth-powder boxes, and the like can be found at the dealers in druggists' glassware and sundries. Whitall, Tatum & Co., Philadelphia, New York, and Boston, have an excellent catalogue of them.

All arrangements for working metals—blow-pipes, furnaces, crucibles—belong with assayers and chemical apparatus. Eimer & Amend, Third Avenue, New York, have a good catalogue of these.

Furnaces are made by the American Gas-Furnace Company, Nassau Street, New York, and their advertisement should be secured, as well as that of the cabinet-makers who made the valuable instrument-cases for Dr. C. F. Ives and Dr. J. Morgan Howe.

Enamel fillings I suppose to be founded on the enamels and colors used by jewellers and in decorating china. A. Sartorius & Co., 28 Barclay Street, New York, give a long list of these.

Forceps, lances, scissors, probes, syringes, and all instruments for oral surgery can be had of dealers in surgical instruments. Tieman & Co., 107 Park Row, New York, publish a large catalogue.

Files, broaches, chucks, steel for matrices, lathes, polishers' supplies, can be had at the dealers in fine tools. Frasse & Co., Park Row, formerly 62 Chatham Street, New York, issue a fine catalogue of them, and so do Montgomery & Co., Fulton Street, New York. A list of all the makers and importers of tooth-brushes, etc., will be of value.

For many years I have had nearly all my instruments made for me. I like a small shop where I can get near my workman. A list of all in New York would be of value. A similar list of the workmen of Philadelphia would add much to the interest of the advertising pages of our journals.

The chemist and assayers have much of interest to us now, but much more can be done by interesting them in our filling-materials and metals. We have united on a series of college text-books, why not unite on a series of college filling-materials? What better work can be done by a society than to take up and bring this matter to the attention of leading chemists and pharmacists?

The makers of fine soaps can also be asked to advertise in our journals, and so can dealers in wax and the gums that enter into modelling compounds. Thus even asbestos and charcoal, crocus and pumice, polishing compositions and whiting, can all be made to contribute to our journals, as we must constantly pay tribute for them.

The names of the chemists who analyze tooth-substances can be put in the list, together with the artists who can make cartoons to illustrate our papers. As hospitals have their formulæ, so every dental school can have its formulæ for gold-plate solders, tooth-bodies, etc. We should not be quite so apt to go to the extreme in advocacy of any one thing, like copper amalgam, if we had a wider discussion of metals. I have never used it, because I heard nothing said in its favor that convinced me that it was as good for a soft, poor tooth as what I had done for years. When called to treat a tooth so poor as to need its tubuli filled, I cauterize with nitrate of silver, and afterwards introduce my amalgam.

In schools at a distance from manufacturing centres, a room could be properly given a workman who was trained to make and repair instruments, regulating appliances, etc. The early years of practice with young men will be made much easier, and their value as assistants increased, if they are trained in the making of appliances.

"Every man to his trade" was an old motto. Every man to his part of a trade is the danger of to-day.

The manufacturing desire to control and the need and benefit of that system is so great in many ways that in some of the watch-factories workmen are employed who can only make one part of a watch, and who may be ignorant of the work done in the next room.

We hear of the "rights of capital," the "rights of labor;" let us hear now of the "rights of occupation,"—the right to learn a trade, the right to engage in business. The Crispins were not given a charter in Massachusetts because they proposed to limit the number of apprentices to be taken. We have not heard that any combination which has the effect to limit the number of persons that can engage in an occupation has obtained a charter there.

The schools for teaching trades have come into being since this exclusive spirit has developed among workmen, exclusive action being met by liberal action.

The time has perhaps not yet come for teaching the making of our instruments as a trade, but some kindred trades could be united in a course at industrial schools. Mould-making for teeth, and die-sinking, surgical instruments and appliances, oral, dental, general, and electrical, would send out men who would not fail of employment, perhaps in some small shop as owner, or often in some school or hospital in any part of the world. Teaching the making of our materials in the schools of pharmacy is also one of the possibilities of the future regarding our resources, and also one of the means for broadening our relations and development.

Dr. Bonwill makes a valuable suggestion,—viz., to cultivate the capacity to improve, that shows itself among students, so that in the greater variety that would result we should have greater freedom from the combination, the Tooth-Crown Company, etc. I am not able to quote his own language, but I think the above is the substance. A list of the various workmen who can do the work required or have done similar work should be kept to make the suggestion practical.

Dealers naturally wish to narrow the channels of trade; we must, in growing, broaden them. They will not keep things for which there is no demand, they may not wish a demand for things they do not wish to keep.

By reporting the names of small makers who serve us well, we would relieve much of the tension.

If those who are interested in alloys will give us a paper on them and publish the name of an assayer or other competent person who has been instructed by them, a channel of information and supply will be opened that will be of value.

Work like this indicated need not be confined to America, but wherever our speciality has a foothold it will ultimately be needed.

The names of firms given are intended merely to indicate some of the lines that can be followed, and because their catalogues can be had by those interested. The same lines can be followed in other parts of the country and much help of influence and value secured. But the activity of professional life is an essential element and sum of all action and will show itself in many ways. To some it may be given to work on committees, to some to edit, to some to improve instruments, to some to study cements, to some to work with the microscope, to some to investigate medicines, to some to encourage and aid, as they can, those who are doing any work for us, but all should be done as for our specialty to aid its development, while it aids to make the perfect man that is to be. It is not for us to say what form professional spirit shall take, or in what work it shall manifest itself, for like love it will go where it is sent by the angels of our nature; but it is for us to make as far as we can the crooked places straight, and the rough ways smooth, that it may enter and dwell in our specialty, that light may not go out from among us and leave our souls in darkness. Are there those who think these questions too small for discussion?

"There is no great and no small
But the soul that maketh all."

